# Treatment of Orchitis, with Note as to Its Varieties, Pathology, Prognosis, Treatment, Etc.

**Published:** 1880-10

**Authors:** G. Halsted Boyland

**Affiliations:** Baltimore, Md.


Vol. I.——*October*, *1880*.——No. 10.

ARTICLE I.

### TREATMENT OF ORCHITIS, WITH NOTE AS TO ITS VARIETIES, PATHOLOGY, PROGNOSIS, TREATMENT, ETC.

BY G. HALSTED BOYLAND, A. M., M. D., ETC., BALTIMORE, MD.

Epididymitis need not necessarily exist alone, as such, and although no less an authority than Dr. Adolp Bardeleben, states that it appears mostly isolated, we are at loss to accept that there is not, as a rule, at least, some sympathetic affection of the testicle itself, an organ between which and the epididymis, so intricate a physiological relation exists. It is easy to understand how the numerous winding of the epididymis must furnish a point of rest for the inflammation to fasten on, when its place of departure was the urethra, or when it has followed along the course of the vas deferens. The swelling is relatively more marked in epididymitis than in any other variety of orchitis. It is rapidly developed and generally shows a great degree of tenacity, leaving for some time after a number of small, hard places in the region of the cauda epididymis; these are to be distinguished from those that appear originally as symptoms of chronic inflammation; such would at once direct our attention to scrofula or syphilis.

*Vaginalitis and Periorchitis* belong to hydrocele.

*Orchitis genina.* Orchitis in the strict signification of the word, or inflammation of the substance of the testicle itself, is perhaps oftener the result of injuries from blows or bruises, than from gonorrhœa. It may also be caused by taking cold. *Pure idiopathic orchitis* which may be also induced by a shock, wound or foreign body may as well be of rheumatic origin.

*Secondary or Sympathetic orchitis* differs from this in that it owes its origin to irritation or inflammation in other organs. Ordinarily the seat of the primary inflammation by means of which such a sympathetic orchitis is developed, is the urethra, the prostate gland or the bladder, and in most all such cases it is an extensive gonorrhœa, reaching as far as the colliculus seminalis, that lies at the bottom of it. *The dyscrasic* inflammation of the substance of the testicle, which evidently rests upon a syphilitic basis as well as tubercle of the testicle, both of which are of chronic nature, are easily distinguished from traumatic orchitis that runs an acute course, and if the wound was severe, suppurates in spite of the most energetic treatment.

In orchitis from gonorrhœa, the pathological process is simply a continuation of the inflammation in the anatomical continuity and contiguity, by means of the ductus ejaculatorius and vas deferens, from the urethra to the testicle.

*Symptoms.* Sometimes (though in those cases of orchitis that have fallen under our own observation, such has not been the case,) symptoms of an affection of the general system, such as malaise, slight nausea, even vomiting, syncope, chills or fever precede the local ones. There is a feeling of heaviness in the scrotum; the pains usually at first down the spermatic cord soon locate themselves in the testicle, which suffers intensely until the third or fifth day, when the pains having attained their most acute point, remain stationary for twenty-four hours; they then gradually ease, disappearing entirely only in from two to three weeks. The swelling of the testicle usually begins after the pains have lasted some time. Sometimes the spermatic cord swells first, generally the epididymis. The rapidity with which the tumor attains its greatest size (in from three to five days) hardly allows to follow closely the process of swelling. The testicle attains often the size of a man's fist: to the touch it is hard, with increased temperature and bright redness of color. *Syphilitic and Scrofulous Swellings of the testicles* are of a somewhat milder type. There is less pain and both testicles are oftener affected. A close inspection of the subjective symptoms in all doubtful cases with an eye to dyscrasia, will generally make us clear as regards syphilitic or scrofulous origin, and then our plan of treatment is fixed. *Prognosis.* The prognosis is not favorable. Although the statement of Ricord, that the testicle is lost every time, is perhaps too sweeping, it must nevertheless be admitted that many testicles are thus rendered sterile and give rise in consequence to physical trouble, that may go as far as deep melancholia.

*Treatment.* Antiphlogistic means are certainly indicated. Orchitis in its mildest form—epididymitis after gonorrhœa—runs a tolerably rapid course and may gradually disappear of itself; but no inflammation of the testicle ought to be left to itself. If the pains are intense and the fever high, ice, leeches to the perineum, and purgatives render good services. The patient must avoid carefully all movement of the diseased part. Only in very light cases can he leave the recumbent position, and then not without wearing a well-fitting suspensory; even in bed, bandages and apparatus ought to be applied to insure rest and elevation to the testicle. Strapping the scrotum with adhesive plaster in order to reduce the inflammation or bring it to a more speedy discussion has long been practiced. The principle is *compression and immobilization.*

Although the certainty of this method in this particular instance is not universally admitted, nevertheless, no one will deny that immobilization and compression, the latter evenly applied and not too severe, are valuable aids in the treatment of orchitis.

If the patient experiences more pain during the application of the bandage and if the pain does not gradually relax, in order to become quite bearable at the expiration of a few hours, it is evident that it is doing no good and must be cautiously removed by thorougly wetting it with warm water. If, on the other hand, the bandage is well borne, the pains not only decrease but the swelling also in such a manner that the bandage becomes loose and must be renewed in a day or two, if it is desirable to continue this treatment. As for my own experience in orchitis, I prefer compresses thrown over the testicle—they should be first wet with a lotion consisting of the following proportions:

| | | |
|---|---|---|
| ℞ | Sulph. Morphia | grs. viii. |
| | Liquor Plumb. subactet. dilut | ℥ i |

M. S. For external use.

It is better to have a large quantity of this, say one pint, put up, and keep the compresses continually wet with it. If the strapping plaster is used it will do better service when prepared of the U. S. Dispensatory officinal compound galbanum. Whatever treatment is adopted the diet should be low and the bowels kept regularly open. For this purpose mild saline purgatives are most cooling. If the disease does not yield we must be ready to treat an acute hydrocele that may result or to open any abscesses that may form, after which we may reasonably look, if not for a radical cure, at least, for a long period of relief for the patient.

The indicatio causalis is of great importance in syphilitic orchitis. Ordinarily, preparations of quicksilver have been used already before the appearance of testicular disease. If this is not the case, by beginning our treatment with them we may attain better and quicker results. Under other circumstances we may give at once iodide of potash, which after previous use of mercurial preparations gives brilliant results. Since the introduction of these drugs numerous testicles have been not only retained but really healed, whereas formerly many were lost by castration. If the word "*isolirt*" (although used in an ambiguous manner by Bardeleben,) means that one testicle only is more frequently affected than both, we are fully in accord with him, although his explicitness is at fault. We hold epididymitis without affection of the orchis to some extent to be of doubtful existence.

